# Rethinking Mandated Drug Treatment: Why Expanding Freedom Requires Structural Drug Policy Reform

**DOI:** 10.1002/hast.70061

**Published:** 2026-05-06

**Authors:** Tim Holland, And Tiffany O'Donnell

**Keywords:** addiction, coercion, critical appraisal, harm, substance use, drug policy, person‐centered care, bioethics

## Abstract

*In this same, May‐June 2026, issue of the* Hastings Center Report, *Brendon Saloner suggests that mandated drug treatment can expand freedom and promote egalitarian social justice so long as three criteria are met. Drawing on our clinical experience in addiction medicine and a critical appraisal of the available evidence, we challenge this claim. We argue that Saloner's three criteria are unlikely to be met within jurisdictions that criminalize drug use and lack supports for people who use drugs. The criterion that mandated treatment be “likely to be beneficial” is undermined by the coercive nature of such interventions and the lack of evidence supporting their effectiveness when compared to voluntary care. Further, the application of mandated treatment risks extending carceral oversight to individuals charged with minor offenses, disproportionately affecting equity‐denied populations. Finally, we question whether infringements on liberty through legal oversight can genuinely expand freedom, particularly when such measures exacerbate social instability and undermine the potential for voluntary engagement with care. We contend that meaningful expansion of freedom for people who use drugs is better achieved through voluntary, community‐based treatment models supported by robust social infrastructure. A radical reorientation of drug policy—away from coercion and toward supportive, person‐centered care—is necessary to achieve the ethical aims of reducing harm, promoting autonomy, and advancing egalitarian social justice*.

## Other Voices

As experienced addiction‐medicine physicians who've supported countless patients through their substance use journey, we greatly appreciate Brendan Saloner's balanced and compassionate consideration of mandated drug treatment in this issue of the *Hastings Center Report*. Saloner argues that mandated treatment can expand freedom and promote egalitarian social justice so long as three criteria are met: “the treatment is likely to be beneficial to a person who is chronically unstable, … the terms of oversight are limited and proportional to the original criminal offense, and … the infringement of liberty involved in legal oversight will help expand the freedom of both the treatment participant and their community.”^1^ However, a deeper analysis of the three criteria reveals some problems.

Although it is difficult to quantify “likely to be beneficial,” we're quite certain that this criterion is nullified by virtue of the treatment's being mandated.^2^ Saloner's single source that provides empirical evidence in support of mandated treatment is a report from the National Institute of Justice.^3^ Our most relevant concern with this document is that it compares people in mandated treatment with people who are incarcerated. Incarceration increases the severity and harms associated with substance‐use disorder,^4^ so it makes sense that drug courts are likely less harmful than incarceration. While this point is well taken, there is a lack of evidence that mandated treatment is “likely to be beneficial” if the comparison involves a more appropriate control group.^5^ What evidence that does exist points toward uncertainty, at best, or harm, at worst.^6^

We also take issue with the notion of the “person who is chronically unstable.” Instability is rarely inherent in the person. Rather, external factors such as childhood trauma, poverty, and housing insecurity are the primary drivers of instability in our patients’ lives, and substance use is often a coping strategy to deal with that chaos. Involvement in the criminal legal system, even for mandated treatment, often exacerbates instability, making future employment and housing harder to reach. By contrast, evidence‐supported voluntary treatment options appealing to the needs of individuals at varying stages of disease would be very welcome alongside the requisite social supports that would truly mitigate instability.

Saloner's second criteria—that the terms of oversight be “limited and proportional to the original criminal offense”—would, we believe, not be applied as justly and humanely as needed. We frequently encounter patients who've been charged with minor drug‐related offences that have not warranted jail time. While charges are sometimes the impetus for individuals to seek out treatment voluntarily and make changes of their own accord, in many cases, people continue on with business as usual, engaging in whatever marginalized behaviors are required to simultaneously have their needs met and stay out of jail in a world that criminalizes their condition. If mandated treatment for addiction were a broad societal strategy, these same patients may have been forced into mandated treatment for such low‐level offenses, thus subjecting them to a carceral sanction that otherwise would have been avoided. Forcibly subjecting people to what is likely to be ineffective treatment at a time when they are not readily seeking help may act as a detriment to voluntary engagement in the future. Moreover, if previous experience with the application of judicial measures can be a predictor of future behavior, we could expect that an expansion of mandated treatment is most likely to fall on equity‐denied groups.^7^

With that in mind, we must focus our attention on the nature of the “original criminal offense,” which is often caught up in our society's unjust treatment of drug use and people who use drugs. For example, public use is often due to the societal constraints of homelessness and a lack of options for safe, discrete consumption. A person struggling with addiction may engage in petty theft to meet their most basic needs—a generally forgivable act providing that someone is sober. Drug possession itself does not cause harm to the person or their community, yet a person may be caught up in the carceral system simply because they are in possession of a drug.

We appreciate that Saloner does not propose to inflict mandatory treatment on every individual who is found to be in possession of an illicit drug. If every judge possessed Saloner's compassion and balance, we would not be worried. However, if you give a child a hammer, all the world becomes a nail. If courts are given the opportunity to mandate treatment, every person who uses drugs becomes a candidate—especially those from equity‐denied groups.^8^

An examination of Saloner's final criterion—that the infringement of liberty that legal oversight entails will help expand the treatment participant's and their community's freedom—draws into question the true purpose of drug policy. What are we trying to achieve with mandated drug treatment (or any drug policy for that matter)? Reduced criminal activity? Sobriety? Happiness? Abstinence? Stability? Saloner describes decriminalization in Portugal as a success, and in Oregon as a failure. If the objective was to reduce the harms associated with problematic substance use, then Portugal has done better than Oregon, but it is unlikely that decriminalization in isolation accomplished this goal. Portugal spent considerable time and resources on community‐based, voluntary treatment opportunities and other supportive social infrastructure prior to decriminalization. Oregon did not. People in recovery will often regard their reduced use of drugs as an expansion of freedom—and under this lens, Portugal did more to expand freedom than Oregon. However, if we consider decarceration an important expansion of freedom, Oregon's decriminalization policy would be a success, as was Portugal's.

The expansion of freedom for people who use drugs and for communities, which could mean reducing the number of people in the carceral system, reducing criminal activity that harms our communities, reducing problematic drug use, or reducing mortality, is an appropriate goal for drug policy. The best way to achieve these aims is through a voluntary, community‐based spectrum of care.^9^ Defining success in addiction treatment is highly individualized, and success is most often achieved through techniques that promote individual freedom through empowering, supporting, and even incentivizing people to reach their own self‐stated goals.^10^ Mandated treatment forces a level of readiness on people while constraining personal freedom and increasing instability in their lives—perhaps the reason that studies have failed to demonstrate clear benefit of mandated treatment for substance use. Saloner's proposition risks pulling more people into the judicial system and *away* from a path that may eventually lead to engaging in effective voluntary options—all under the guise of helping them for their own sake.

Saloner proposes that his arguments would move us to a “just right” place between what punitive drug‐policy hawks push for and full drug decriminalization. We agree that drug courts are preferable to punitive incarceration, but, when considering the available evidence on addictions treatment, his arguments push us beyond drug courts and into a realm of decriminalization, or even legalization—provided that this progressive realm is associated with the requisite voluntary treatment resources and protective regulations. We respect that Saloner intends for his arguments to be taken within the constraints of current U.S. drug policy and underresourced social support systems. However, if we're restricting our solutions to these unjust limitations, we're merely rearranging the deck chairs on the Titanic.
